# A Solitary Duodenal Metastasis from Transverse Colon Cancer Detected Six Years after Curative Resection: A Case Report

**DOI:** 10.70352/scrj.cr.25-0579

**Published:** 2026-01-24

**Authors:** Shingo Otsuji, Hiroki Shimizu, Jun Kiuchi, Taisuke Imamura, Kenji Nanishi, Tomohiro Arita, Toshiyuki Kosuga, Yusuke Yamamoto, Hirotaka Konishi, Ryo Morimura, Hitoshi Fujiwara, Atsushi Shiozaki

**Affiliations:** Division of Digestive Surgery, Department of Surgery, Kyoto Prefectural University of Medicine, Kyoto, Kyoto, Japan

**Keywords:** colon cancer, duodenal metastasis, late recurrence

## Abstract

**INTRODUCTION:**

Recurrence of colon cancer after 5 years or longer from primary surgery is relatively rare and furthermore, isolated metastasis to the duodenum is extremely rare.

**CASE PRESENTATION:**

The patient was a 79-year-old male. He underwent laparoscopic partial colectomy with radical lymphadenectomy for transverse colon cancer. Pathohistological examination revealed that the tumor stage was T4aN0M0, resulting in R0 resection. An oral adjuvant chemotherapy was administered only one course due to side effects. The serum carcinoembryonic antigen level began to increase after 5 years and 1 month postoperatively. An upper gastrointestinal endoscopy at 6 years postoperatively revealed a submucosal tumor at the inferior duodenal angle, and subsequent endoscopic ultrasound-fine needle aspiration confirmed the diagnosis of duodenal metastasis from transverse colon cancer. As no other metastatic lesion was detected, open partial duodenectomy was undergone and R0 resection was achieved. The patient has been under regular follow-up without adjuvant chemotherapy and has survived without recurrence for 2 years since the second surgery.

**CONCLUSIONS:**

Although extremely rare, the possibility of isolated duodenal recurrence after surgery for colon cancer exists. With a brief review of the literature, we report here a rare recurrence case of transverse colon cancer which was discovered at 6 years postoperatively and resulted in curative resection.

## Abbreviation

CEA
: carcinoembryonic antigen
UICC
: Union for International Cancer Control

## INTRODUCTION

Recurrences of colorectal cancer 5 years or longer after curative surgery for primary lesion are relatively rare, occurring in less than 5% of cases.^1)^ Furthermore, isolated metastasis to the duodenum is almost unheard of. Here, we report an extremely rare case of an isolated duodenal metastasis occurring 6 years after primary surgery for transverse colon cancer, which was successfully treated with curative resection. A brief review of the literature is also provided.

## CASE PRESENTATION

**Fig. 1:**
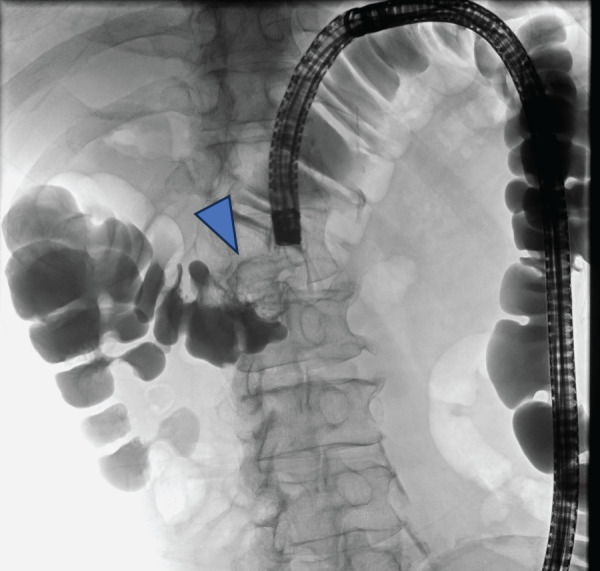
The tumor was present in the transverse colon (blue triangle).

**Fig. 2:**
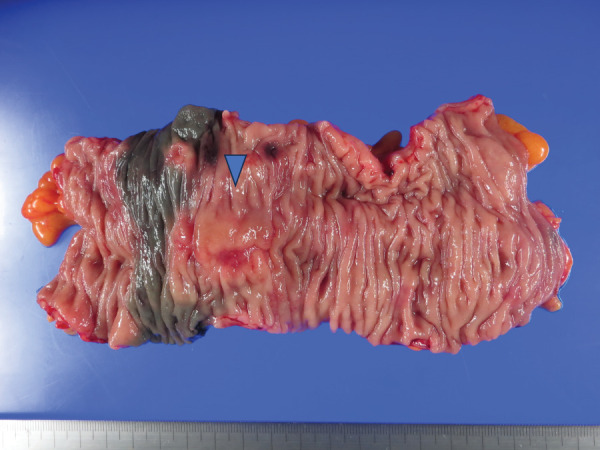
The specimen of the initial surgery. The pathological examination of the resected specimen revealed that staging of the tumor (blue triangle) was T4aN0M0.

The patient is a 78-year-old male with no notable past medical history or family history. He underwent laparoscopic partial colectomy with radical lymphadenectomy for transverse colon cancer. **Figure 1** shows the location of the tumor. The pathological examination of the resected specimen (**Fig. 2**) revealed that the tumor staging was T4aN0M0 (UICC 8th Edition) with the following histological findings: well differentiated with a component of poorly differentiated adenocarcinoma (tub1 > por2), presence of minimal lymphatic invasion (Ly1a), presence of extramural perineural invasion (Pn1b), and negative for any margins according to the Japanese Classification of Colorectal Carcinoma.^2)^ Adjuvant chemotherapy with Tegafur–Uracil combined with Leucovorin was initiated postoperatively, but it was discontinued after one course due to side effects, and the patient was placed under observation.

**Fig. 3:**
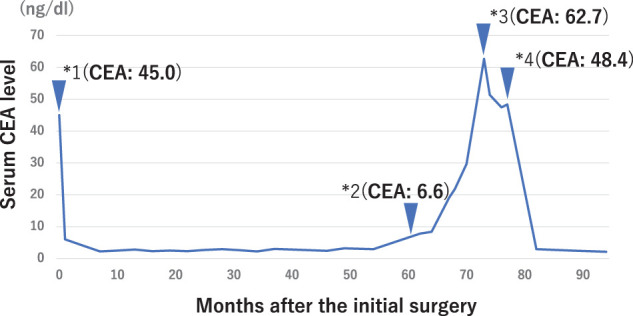
The trends of serum carcinoembryonic antigen. Each point represents as follows: Point 1: the initial surgery. Point 2: the serum carcinoembryonic antigen (CEA) level began to increase at 5 years and 1 month post-surgery. Point 3: the serum CEA level reached at the maximum at 6 years and 2 months post-surgery. Point 4: the second surgery.

**Fig. 4:**
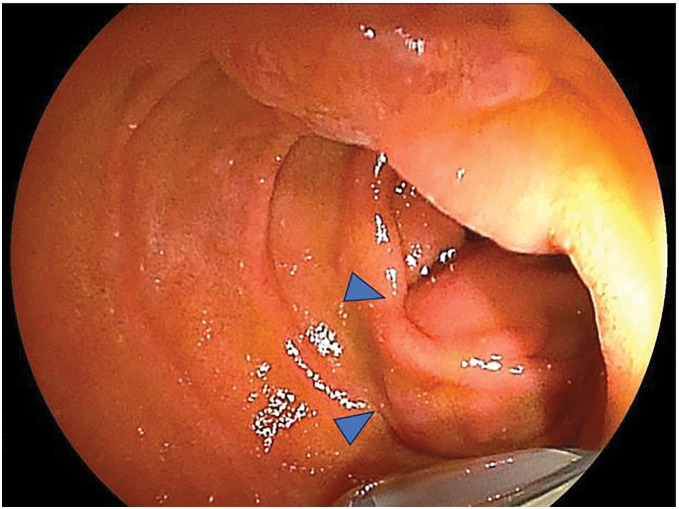
Preoperative upper gastrointestinal endoscopy examination. The submucosal tumor (blue triangles) was found in the duodenum.

**Fig. 5:**
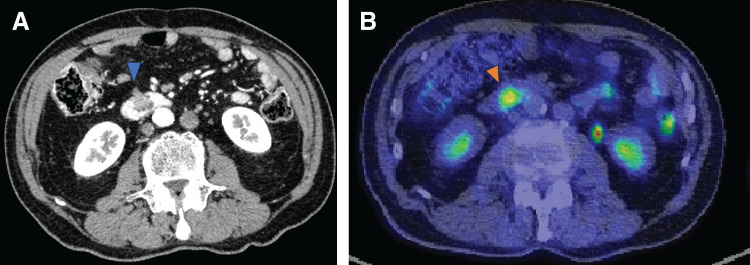
Preoperative findings of imaging examinations. A soft tissue shadow (blue triangle) was found at the duodenum on CT (**A**) and it (red triangle) showed strong accumulation of fluorodeoxyglucose on PET–CT scan (**B**).

**Fig. 6:**
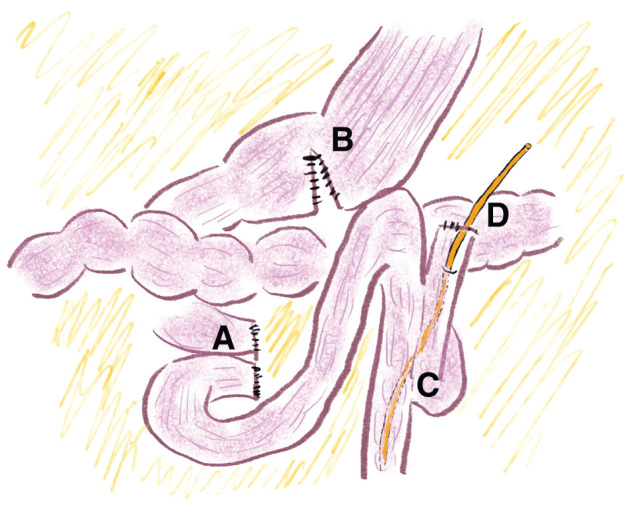
Schematic illustration after reconstruction. (**A**) The jejunum was transected 20 cm distal to its origin, and the distal stump was anastomosed to the proximal duodenal stump. (**B**) A gastrojejunostomy bypass was performed using Devine anastomosis. (**C**) An anastomosis was created between the efferent limb and the jejunum on the Treitz ligament side. (**D**) A feeding jejunostomy tube was inserted through the jejunal stump.

**Fig. 7:**
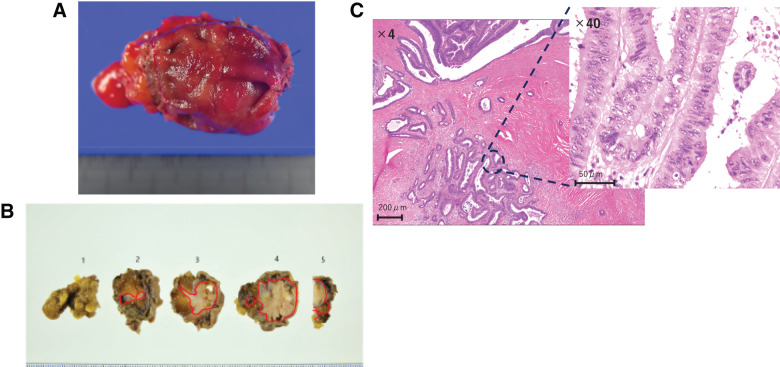
Surgical specimen and pathological images of the second surgery. (**A**) The resected specimen. (**B**) The red line shows a margin of the tumor. The resection margins were negative. (**C**) The findings of Hematoxylin-Eosin staining.

The patient completed the prescribed 5-year postoperative follow-up period, and it was planned to end his follow-up at our department. However, at 5 years and 1 month postoperatively, a mild increase in serum CEA levels (6.6 ng/dL) was noted. The chronological changes in CEA levels since the primary tumor resection are shown in **Fig. 3**. The patient initially refused additional examinations and was only observed, but due to the continued increase in CEA levels, he agreed to receive additional examinations. The endoscopy at 6 years postoperatively revealed a 20-mm submucosal tumor at the inferior duodenal angle, opposite from papilla of Vater (**Fig. 4**). For further investigation, endoscopic ultrasound-fine needle aspiration was performed and the tumor was pathologically diagnosed as adenocarcinoma, leading to the possible duodenal metastasis from previously resected transverse colon cancer. CT imaging revealed an 18-mm soft tissue shadow from the descending to the horizontal part of the duodenum (**Fig. 5A**). PET–CT showed abnormal fluorodeoxyglucose uptake in the duodenum corresponding to the tumor location (SUVmax: 4.27) (**Fig. 5B**). No metastatic lesions were detected in other organs. Based on these findings, the patient was diagnosed with isolated duodenal metastasis from transverse colon cancer, and underwent partial duodenectomy with duodenojejunostomy, gastrojejunostomy bypass, and creation of a jejunostomy (**Fig. 6A**–**6D**). Pathological examination of the resected specimen revealed an atypical columnar epithelium with enlarged nuclei, exhibiting irregular tubular and papillary structures proliferating from the submucosa to the subserosa of the duodenum, leading to the diagnosis of duodenal metastasis from transverse colon adenocarcinoma. The resection margins were negative, achieving curative resection (**Fig. 7A**–**7C**).

The patient was discharged on POD 83 due to postoperative complications, including pulmonary vein thrombosis and aspiration pneumonia. No recurrence was observed without adjuvant therapy in 2 years since the second surgery.

## DISCUSSION

**Fig. 8:**
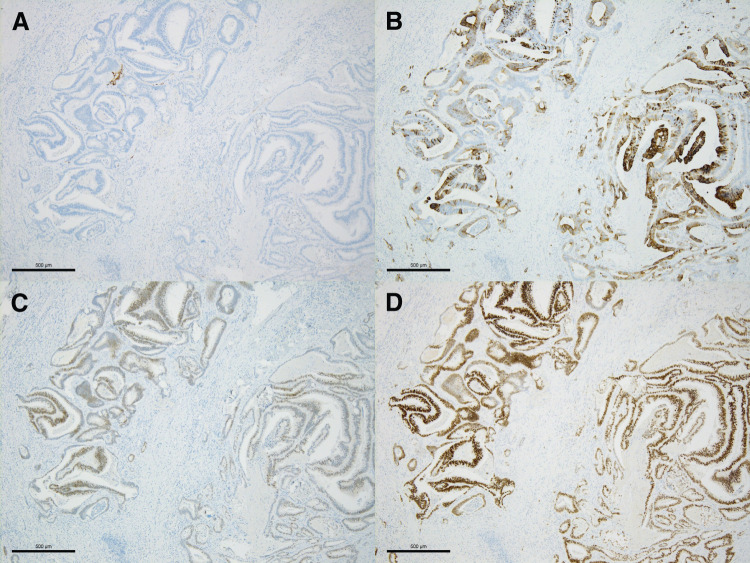
Immunohistochemical staining of the resected duodenal specimen, original magnification ×4. The tumor was observed as negative with CK7 staining (**A**), and positive staining with CK20 (**B**), CDX2 (**C**), and SATB2 (**D**) staining.

This case must be extremely rare due to the late recurrence occurring later than 5 years post-surgery, the isolated duodenal metastasis from colon cancer, and the successful curative resection for the recurrent lesion with 2-year disease-free survival. First, regarding the interval until recurrence, approximately 90% of postoperative recurrence of colorectal cancer recurrences occur within 3 years of post-surgery, with less than 5% recurring after 5 years.^3)^ Therefore, treatment guidelines generally set the postoperative surveillance period to 5 years. For instance, according to the National Comprehensive Cancer Network Guidelines, CEA measurements are recommended every 3–6 months for the first 2 years post-surgery, and every 6 months for the subsequent 3 years, totaling 5 years of follow-up for the patients with Stage II to III colorectal cancer.^1)^ Regular CEA measurements and imaging examinations beyond 5 years post-surgery are not recommended.^1)^ In this case, we continued follow-up beyond 5 years post-surgery due to the slight increase of tumor marker and could detect the recurrence in a relatively early phase. As a result, we could perform a curative resection on the recurrent lesion, which has contributed to the current disease-free survival. Tumor dormancy may be involved as one of the mechanisms contributing to the late recurrence observed in the present case. Tumor dormancy is defined as a state in which disseminated tumor cells remain viable but non-proliferative for a prolonged period and subsequently become reactivated, leading to recurrence or metastasis through various pathways.^4)^ In this case, despite a relatively advanced depth of invasion of the primary tumor (T4a), no evidence of recurrence was observed during the standard 5-year postoperative surveillance period, and recurrence was detected only thereafter. Therefore, the concept of tumor dormancy is not inconsistent with the clinical course observed in this case. Another rare aspect of this case is that the site of metastasis was the duodenum. The common sites of distant metastases of colon cancer are the liver, lung, and peritoneum, followed by the bone, brain, thyroid, and adrenal gland.^3)^ However, metastasis to the duodenum, as seen in this case, is extremely rare and scarcely reported. A PubMed search using “duodenal metastasis” and “colon cancer” revealed only two case reports, except our case (**Table 1**).^3,5)^ In two cases, including our case, the recurrent site was isolated at the duodenum and the period from primary surgery to recurrence was more than 5 years. The possible mechanisms to explain how metastasis to the duodenum occurs are that lymphatic flow from the right-sided colon runs near the duodenum, and the mesocolon of the hepatic flexure is directly adjacent to the duodenum^6)^ The tumor location of primary colon cancer was at the right-sided colon in all cases, which is consistent with this theory. In particular, in the present case, we believe that the presence of minimal lymphatic invasion in the primary tumor, along with the immunohistochemical findings of the duodenal lesion (CK7−, CK20+, CDX2+, and SATB2+) (**Fig. 8A**–**8D**), is compatible with the lymphatic spread of transverse colon cancer to the duodenum. Metastatic lesions at the duodenum typically retain submucosal tumor-like characteristics, and the endoscopic examination is useful for differentiating it from primary duodenal cancer. At the early phase, cases without any symptoms are quite common; however, as the tumor grows, symptoms such as vomiting, nausea, obstructive jaundice, and gastrointestinal bleeding become apparent.^7,8)^ As shown in **Table 1**, the two cases in previous reports had symptoms with the progressing stage of duodenal metastasis, while our case had no symptoms and the recurrence was discovered relatively early due to the increase of tumor marker, which resulted in the only case achieving curative resection for the recurrence. Finally, although long-term surveillance in the present case ultimately led to a favorable outcome as described above, this represents an exceptionally rare situation. Surveillance beyond the duration recommended in current treatment guidelines should therefore be considered only for high-risk patients and further investigation with a larger accumulation of cases is necessary.

**Table 1:** Summary of case reports of duodenal metastasis after colon cancer surgery

No.	Authors	Age	Sex	Primary tumor location	Stage	Time to recurrence	Chief complaint at recurrence	Treatment for recurrence	Simultaneous recurrent site
1	Brahmbhatt et al.^3)^	54	F	Ascending colon	IIIC	1 year and 3 months	Nausea	Best supportive care	Brain, lung, mediastinum
2	Rosado Dawid et al.^5)^	82	F	Cecum	IIIB	10 years	Jaundice	Chemotherapy	None
3	Our case	78	M	Transverse colon	IIB	5 years and 1 month	None	Curative surgery	None

## CONCLUSIONS

We experienced an extremely rare case of curative resected isolated duodenal metastasis at the sixth year after surgery for transverse colon cancer.
